# Incidence of low birth weight in Mexico: A descriptive retrospective study from 2008–2017

**DOI:** 10.1371/journal.pone.0256518

**Published:** 2021-09-10

**Authors:** Mónica Ancira-Moreno, Eric Monterrubio-Flores, Sonia Hernández-Cordero, Isabel Omaña-Guzmán, Isidro Soloaga, Fabián Torres, Moisés Reyes, Yohali Burrola-Mendez, Ariana Morales-López

**Affiliations:** 1 Departamento de Salud, Universidad Iberoamericana, Ciudad de México, México; 2 Observatorio Materno Infantil (OMI), Universidad Iberoamericana, Ciudad de México, México; 3 Centro de Investigación en Nutrición y Salud, Instituto Nacional de Salud Pública, Cuernavaca, México; 4 Departamento de Salud, Universidad Iberoamericana, Ciudad de México, México; 5 Doctorado en Ciencias Biológicas y de la Salud, Universidad Autónoma Metropolitana, Mexico City, México; 6 Departamento de Economía y GEOLab-IBERO, Universidad Iberoamericana, Ciudad de México, México; 7 Centro de Estudios en Computación Avanzada, Universidad Nacional Autónoma de México, Ciudad de México, México; 8 GEOLab-IBERO, Universidad Iberoamericana, Ciudad de México, México; 9 School of Rehabilitation, Faculty of Medicine, Université de Montréal, Montréal, Québec, Canada; 10 CHU Sainte-Justine Research Center, Montréal, Québec, Canada; 11 Maestría en Nutrición Aplicada, Universidad Iberoamericana, Ciudad de México, México; University of Southampton, UNITED KINGDOM

## Abstract

According to the WHO, low birth weight (LBW) affects 15–20% of newborns worldwide. In Mexico, there are no national, state, nor municipal estimates that inform the country’s situation over time. The purpose of this study was to estimate the incidence of LBW at the national, state, and municipal levels from 2008 to 2017, and to estimate the LBW incidence based on maternal sociodemographic characteristics, prenatal care and marginalization indexes at the national level using open national data. We used spatial data analysis to georeferenced LBW incidence at the three levels of geographical disaggregation studied. At the national level, the incidence of LBW increased progressively from 6.2% (2008) to 7.1% (2017), and the country’s capital represented the area with the highest incidence. Southeastern and central states reported the highest LBW regional incidence. At the municipal level, the number of municipalities with an incidence of LBW ≥8% increased in both male and female newborns. The incidence of LBW was higher as the marginalization indexes increases. The results from this study may assist in the identification of vulnerable groups and the development of public health programs and policies with an intersectoral approach that improves maternal and child nutrition.

## Introduction

The World Health Organization (WHO) defines low birth weight (LBW) as weight at birth less than 2500 grams (g) (5.5 pounds) [1]. LBW is considered a global indicator of healthcare delivery, poverty, nutrition, maternal health, and an important predictor of neonatal mortality and morbidity [1, 2]. Neonates with LBW have more than 20 times greater risk of dying than heavier babies [3] and an increased risk of hospitalizations due to diarrhea and respiratory infections in the first year of life [4, 5]. Long-term neurodevelopment consequences associated with LBW include impaired language development [6] and deficits in cognition, attention, and neuromotor functioning [7, 8]. Additionally, LBW increases the risk of noncommunicable diseases such as diabetes and cardiovascular disease [9, 10].

Globally, more than 20 million newborns are LBW infants, which represents 15–20% of all births annually [1]. The prevalence of LBW varies considerably across countries and regions; however, low- and middle-income countries (LMICs) account for a disproportionate burden of LBW [1, 2, 8]. Nevertheless, LBW is a global concern not limited to less-resourced settings. Some high-income countries such as the United States and the United Kingdom still report high rates of LBW (USA incidence of LBW in 2019: 8.3% [11]; UK incidence of LBW in 2015: 7% [9]), especially among vulnerable populations (e.g. populations with low income, indigenous and populations from rural regions).

In 2012, the WHO established a comprehensive implementation plan on maternal, infant, and young child nutrition that includes among its six global nutrition targets, a 30% reduction in LBW by 2025 [12]. To reach this target, Member States need to develop cost-effective interventions at the policy, health system, and community level and through an intersectoral approach [1, 12]. LMICs face particular challenges related to limited and unreliable data and barriers to monitoring programs. In Latin America and the Caribbean, UNICEF estimated 9% of LBW and 17% of births not being weighted, and those weighted are not always measured, recorded, or reported accurately [7]. Mexico, as a Member State, is interested in achieving the six global nutrition targets established by the WHO for 2025.

Mexico is a country in the southern part of North America that is constituted by 32 states. Each state is comprised of municipalities; the country has 2,469 in total. Mexico City is the country’s capital and is constituted by sixteen territorial demarcations [13]. In 2008, the Mexican government established the mandatory and official use of birth certificates that include a full profile of the newborn’s and mother’s health and socio-demographic characteristics. Additionally, the government created a specific task force, the Subsystem of Information about Births (SINAC for its acronym in Spanish), in charge of integrating information about the births occurred in the country, protecting children’s rights and supporting the planning, allocation of resources and evaluation of maternal and child health programs [14]. According to local regulations such as the Norma Official Mexicana NOM-007-SSA2-1993 [15], all newborns must be weighed, their data documented in the clinical file and their weight included in the birth certificate.

Despite the NOM-007-SSA2-1993, there are no national nor systemic reports that estimate and monitor LBW incidence or prevalence. Few studies have calculated LBW prevalence in regional hospitals with a small sample size and a short period analyzed (i.e. one year) [16–18]. In 2013, Buekens, et al. [19] conducted a retrospective study that reported national LBW incidence in 2008 and 2009, with state-level analysis in 2009. Although this study represents the first effort towards a national awareness of LBW, the short period analyzed, the absence of municipal level data, and the lack of information on maternal health variables and sociodemographic characteristics represent important limitations that need to be addressed to fully understand the national situation and develop programs to address this public health problem.

The purposes of this study are: (1) to estimate the incidence of LBW in Mexico at the national, state, and municipal level between 2008 to 2017; (2) to estimate the incidence of LBW based on maternal sociodemographic characteristics, prenatal care, and marginalization indexes at the national level; and (3) to georeferenced the data obtained using spatial analysis. This study would provide a further understanding of the Mexican situation with LBW and assist in the development and implementation of strategies to reach the global nutrition target of a 30% reduction in LBW by 2025.

## Materials and methods

### Study design

This study used a descriptive retrospective design to estimate the incidence of LBW in Mexico from 2008–2017 using national administrative open-access datasets. This period was selected because it represents, up to the development of this research project, the most recent data available. We accessed the datasets and extracted the data that met the inclusion criteria (i.e., live births, gestational age at birth >21 gestational weeks). We excluded data from newborns with missing birth weight and sex.

### Data sources

#### Subsystem of Information about Births (SINAC)

Birth certificates data were accessed from the SINAC website [20]. The certificates include information about newborns’ attributes, maternal sociodemographic characteristics (e.g. age, parity, educational level) and prenatal care [21].

#### National Population Council (CONAPO)

Mexican population census is conducted every 10 years. We used the 2010 Census report to obtain marginalization indexes and area of residence at the municipality, state, and national levels. This information was used across the studied period (2008–2017) [22]. Based on the size of the population, rural areas were classified as with < 2500 inhabitants while semi-urban and urban ≥ 2,500 inhabitants [23]. The marginalization index is a national indicator that differentiates geographic regions according to the global impact of the deficiencies suffered by the population, as a result of the lack of access to education, public services, low income and residence in small localities. CONAPO obtained this index through a principal component analysis that included nine variables (socioeconomic indicators). Subsequently, the stratification was made into five marginations grades [24]. Lower index levels indicate better conditions while high levels worst conditions [24].

#### National Institute of Statistic and Geography (INEGI)

The INEGI developed an open-access national Geostatistical Framework represented by polygons that constitute the spatial limits of all country’s geographic units [25]. The geographic units are coded and are used across all national datasets, such as birth certificates and the population census. This feature allowed us to merge datasets using the same coding system and georeferenced the data.

### Data analysis

For categorical variables (i.e., age groups, education, marital status, health care provider, parity, area of residency, marginalization index, prenatal care, newborn’s sex, gestational age at birth) proportions were calculated; for continuous variables (i.e., maternal age, weight at birth, length at birth,) means and standard deviations (SD) were estimated. Birth weight data that laid ± 3 SD from the mean were considered outliers and removed from the analysis.

#### National, state, and municipal LBW incidences

LBW incidence was estimated by dividing the total number of newborns with LBW by the total number of births that occurred per year at each geographic level: municipal, state and national.

LBW incidences were compared from 2008 to 2017 by estimating the relative risks (RR) using Poisson regression models with Bootstrap estimations with one thousand repetitions to identify statistically significant changes. In these models, we tested the possible interaction between newborns’ sex and year to identify if the change in LBW incidence was differential according to sex. Derived from the interactions, models stratified by sex were carried out, at the national and state level. For all calculations, the statistical significance level was set at 0.05 alpha level except for interaction in which it was 0.1. All analyses were performed using Stata Software 13^®^.

#### LBW incidence based on maternal characteristics

To distinguish LBW incidence based on maternal sociodemographic, prenatal care and marginalization index, the total newborns with LBW per category were divided by the total number of births that occurred in the same category. The marginalization index and area of residency were imputed from the 2010 Census and used throughout the study period.

#### Spatial data analysis

To georeferenced the incidence of LBW we performed spatial data analysis. The Geostatistical Framework dataset from INEGI and the dataset including LBW incidence were imported into QGIS^®^ and merged using the country’s geographic coding system to conduct the spatial data analysis and to create the maps. QGIS^®^ is a free, open-source, software of geographic information system that includes tools for spatial data analysis [26].

## Results

A total of 21,146,302 birth records from 2008 to 2017 were identified. We excluded 1,247,874 observations with missing birth weight data (5.9%) and 244 observations with gestational age <21. In addition, we excluded 20,639 data (which represents 0.01% of the data with birth weight information and a gestational age>21) due to missing information about newborn sex. By excluding this data, outliers were also excluded. We retained for analysis 19,877,545 birth records, 51% of them corresponded to male newborns and 49% to female newborns. Most of the births occurred in a health institution (98.25%) while only 1.31% occurred in a household, 0.04% in public roads, 0.39% in a place other than the public road or home and 0.01% did not specify.

Regarding maternal and newborn’ characteristics from 2008 to 2017, the distribution of maternal age and parity was similar in both periods. In 2017, a higher level of maternal education, an increased of Seguro Popular coverage of 28.2 percentage points (pp) and an increase of 1.4 pp of prenatal care access was observed when compared with 2008. Regarding newborn characteristics, these were similar in both years (S1 Table).

During the study period, it was observed that birth weight distribution showed a displacement to the left for both males and females from 2008 to 2017 (S1 Fig). In females, there was a decrease of 454.5 g in birth weight from 2008 to 2017, whereas for males the decrease was 41.2 g in the same period.

### LBW incidence at national, state and municipal levels

**Fig 1:**
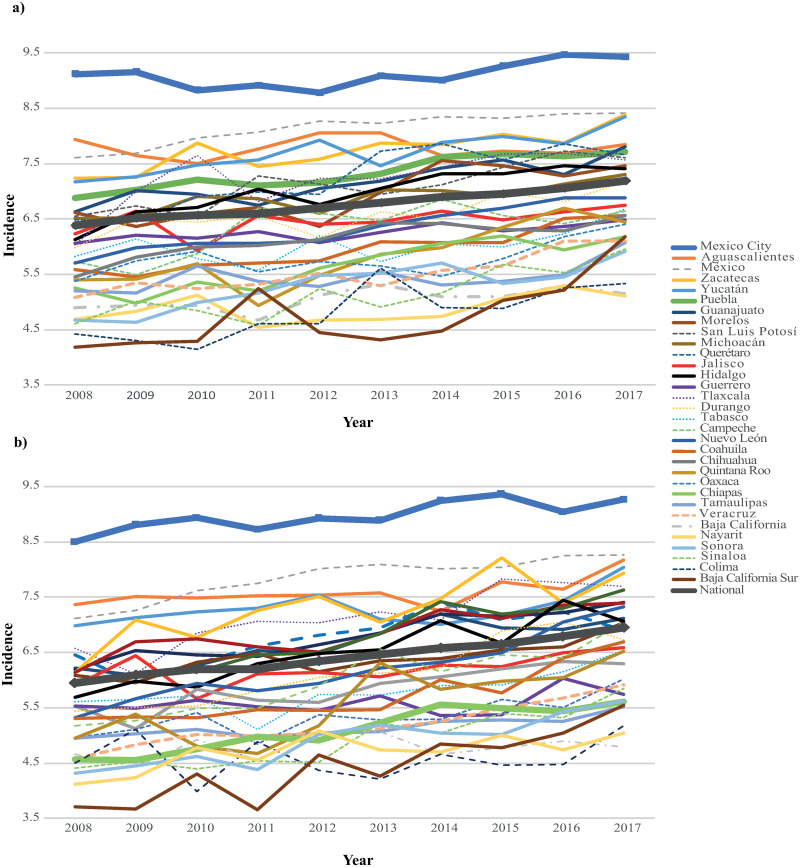
**a.** LBW incidence in males from 2008 to 2017. **b.** LBW incidence in females from 2008 to 2017.

At the national level, a constant increase in the LBW incidence between the years 2008 and 2017 was observed in males and females (Fig 1a and 1b). In 2008, the national incidence of LBW was 6.2% (n = 114,484) and in 2017 was 7.1% (n = 137,985). Stratifying by newborn’ sex, in 2008, this incidence was 6.4% for males (n = 60,981) and 6.0% for females (n = 53,505) while in 2017 these values increased to 7.2% in males (n = 72,342) and 7.0% in females (n = 65,647). The increase was statistically significant for both males and females (p<0.0001). At the state level, a growing but not constant trend was observed.

**Fig 2:**
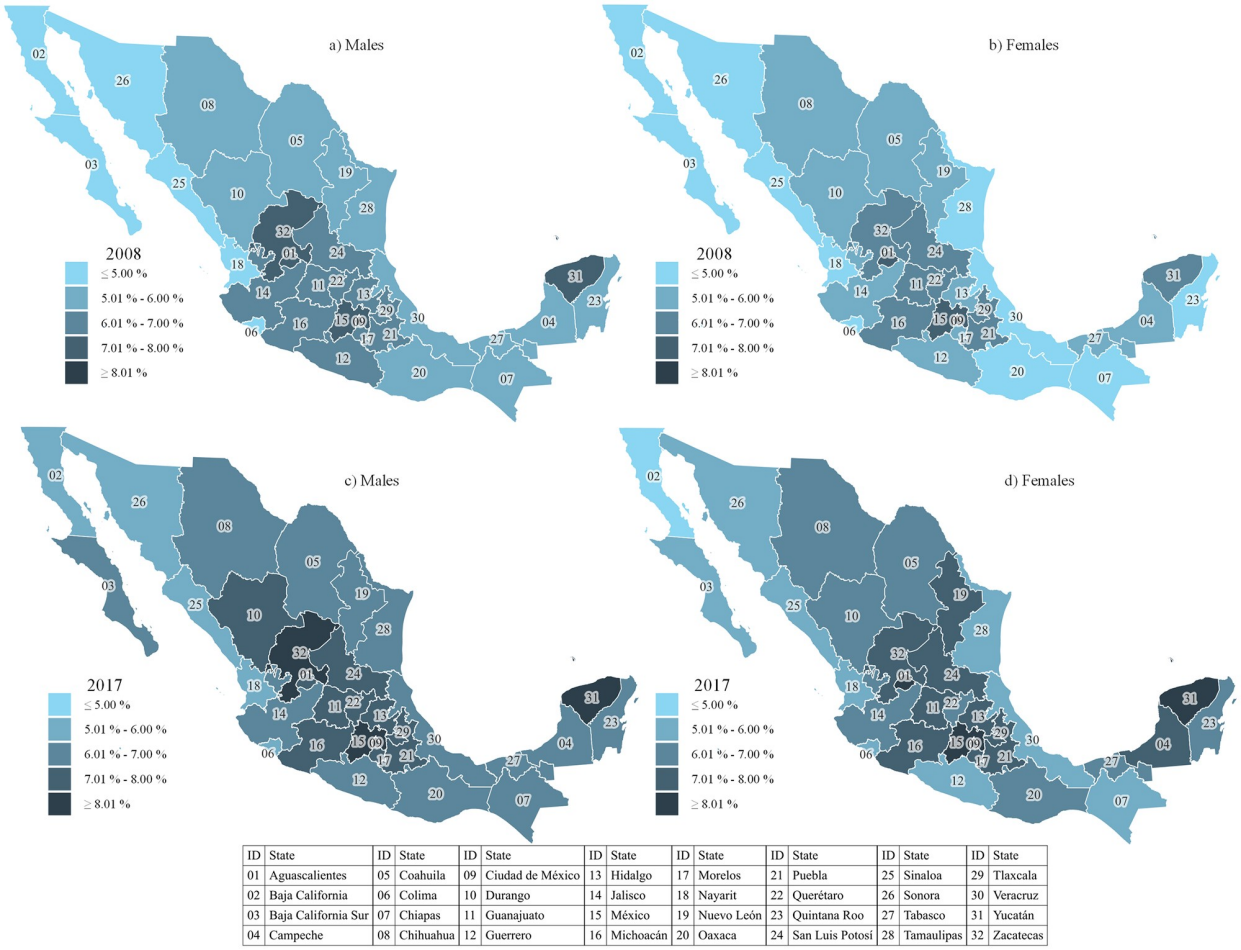
**a.** LBW incidence in live births in Mexico in males, 2008. **b.** LBW incidence in live births in Mexico in females, 2008. **c.** LBW incidence in live births in Mexico in males, 2017. **d.** LBW incidence in live births in Mexico in females, 2017.

Fig 2a–2d represent the maps from the spatial data analysis at the state level by newborns’ sex to visualize the changes between 2008 and 2017. The states with the highest incidence of LBW changed according to sex in both periods. For males and females in 2008, the three entities with the highest index were Mexico City (ID 09) (9.12% and 8.50%, respectively), Aguascalientes (ID 01) (7.93% and 7.37%, respectively), and Mexico State (ID 15) (7.60% and 7.12%, respectively); the fourth and fifth position in males were Zacatecas (ID 32) (7.24%) and Yucatán (ID 31) (7.18%), whereas in females were Yucatán (ID 31) (6.99%) and Tlaxcala (ID 29)(6.57%). In 2017, Mexico City (ID 09) represented the state with the highest incidence (9.43% and 9.26% in male and female, respectively), followed by Mexico State (ID 15) (8.42% and 8.27% in male and female, respectively); for males, the third position was occupied by Zacatecas (ID 32) (8.39%), followed by Yucatán (ID 31) (8.35%) and Aguascalientes (ID 01) (7.85). For females, the third position was Aguascalientes (ID 01) (8.18%) followed by Yucatan (ID 31) (8.04%) and Zacatecas (ID 32)(7.93%).

**Fig 3:**
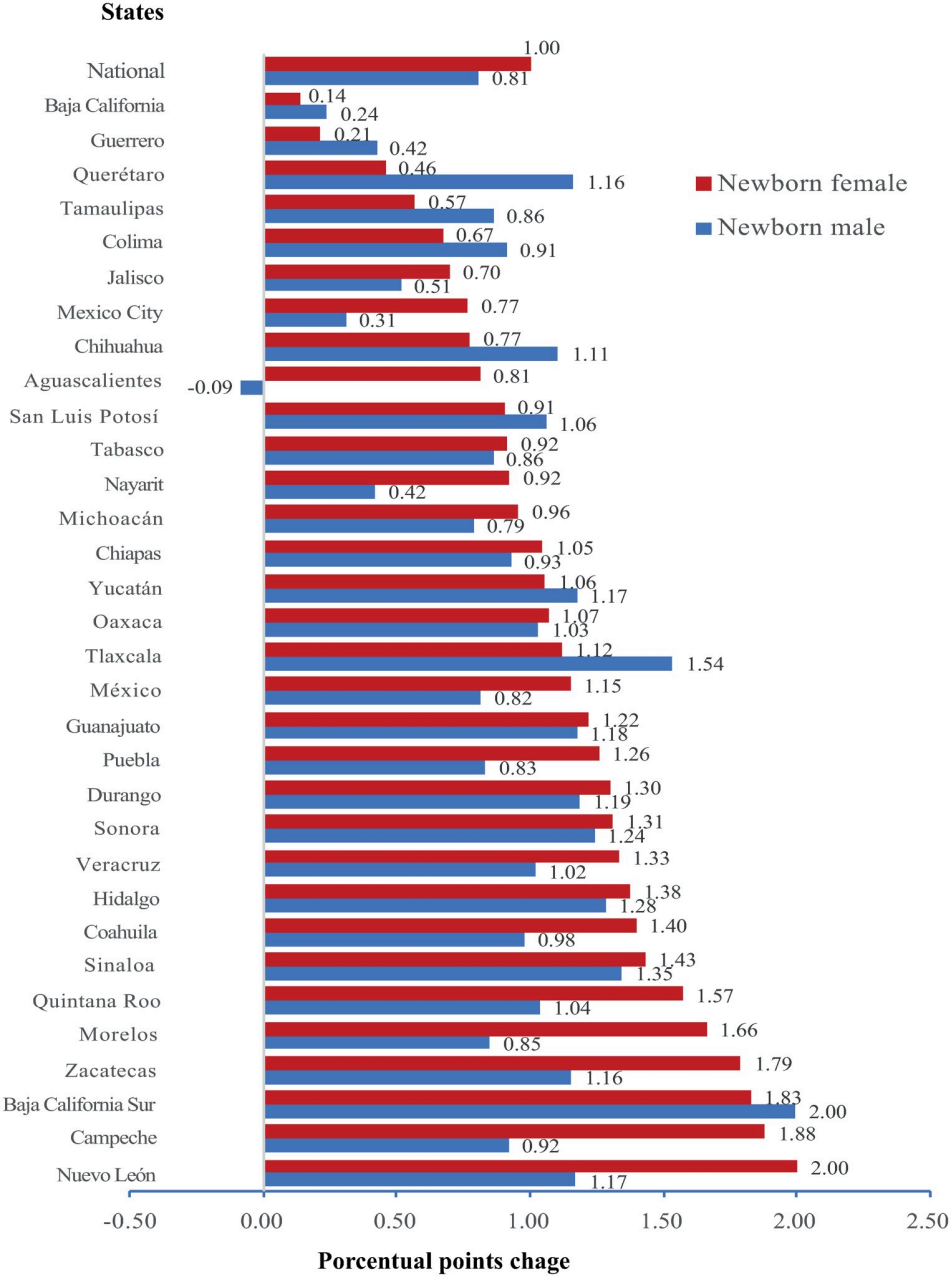
Change in LBW incidence between 2008 and 2017, at national and state levels.

At the national level, there was a statistically significant increase of 1.0 and 0.8 pp for females and males, respectively, from 2008 to 2017 (p<0.0001 in both cases) (Fig 3). At the state level, LBW incidence increased in all states, except in Aguascalientes where there was a decrease in newborn males, not statistically significant. In six states, the change in the incidence was distinct between newborns’ sex. For example, in Aguascalientes (Males: p = 0.007 vs females: p = <0.0001), Baja California (Males: p = 0.07 vs females: p<0.01) and Mexico State (Males: p = 0.18 vs females p = 0.002) the change was statistically significant in females but not in males, whereas in Jalisco (Males: p = 0.02 vs females: p = 0.10) and Sonora (Males: p = <0.0001 vs females: p = 0.08) the change was statistically significant in males but not in females. In twenty-four states, the increase in the incidence of LBW in both sexes was statistically significant while in only 2 states, Guerrero (Males: p = 0.23 vs females: p = 0.48) and Nayarit (Males: p = 0.05 vs females: p = 0.31) the change was no statistically significant. The state with the highest increase in the incidence of LBW in males was Baja California Sur (2.00 pp, p<0.0001); whereas in females was Nuevo León (2.00 pp, p<0.0001) (Fig 3).

The change in LBW incidence from 2008 to 2017 was 13% in males and 17% in females (p<0.0001 for both cases) (Table 1). This change varies by state and only Baja California did not show a significant increase in the RR. At the national level, the State of Mexico, Morelos, Nuevo Leon, Veracruz and Zacatecas, the RR in females was higher than in males (Interaction sex * year, p < 0.1).

**Table 1:** Relative Risk (RR) and 95% confidence intervals (CI) of LBW per year of birthday and State, from 2008 to 2017.

	Male		Female		Interaction sex*year 2008–2017**
	RR (IC95%)	p-value*	RR (IC95%)	p-value*	p-value
**National**	1.13 (1.11, 1.14)	**<0.001**	1.17 (1.15, 1.18)	**<0.001**	**<0.001**
**States**					
Aguascalientes	1.06 (0.97, 1.15)	0.171	1.10 (1.00, 1.22)	0.041	0.525
Baja California	1.09 (0.98, 1.20)	0.103	0.98 (0.88, 1.10)	0.691	0.193
Baja California Sur	1.50 (1.22, 1.84)	**<0.001**	1.40 (1.12, 1.76)	**0.003**	0.659
Campeche	1.15 (1.01, 1.31)	**0.034**	1.31 (1.14, 1.50)	**<0.001**	0.198
Coahuila De Zaragoza	1.17 (1.09, 1.25)	**<0.001**	1.26 (1.18, 1.35)	**<0.001**	0.118
Colima	1.24 (1.04, 1.48)	**0.017**	1.22 (1.02, 1.46)	**0.033**	0.879
Chiapas	1.15 (1.09, 1.22)	**<0.001**	1.21 (1.14, 1.29)	**<0.001**	0.275
Chihuahua	1.16 (1.07, 1.24)	**<0.001**	1.16 (1.07, 1.25)	**<0.001**	0.946
Mexico City	1.08 (1.05, 1.11)	**<0.001**	1.12 (1.08, 1.15)	**<0.001**	0.118
Durango	1.21 (1.12, 1.31)	**<0.001**	1.28 (1.17, 1.40)	**<0.001**	0.354
Guanajuato	1.20 (1.15, 1.25)	**<0.001**	1.20 (1.15, 1.26)	**<0.001**	0.889
Guerrero	1.07 (1.01, 1.14)	**0.025**	1.07 (1.01, 1.14)	**0.034**	0.925
Hidalgo	1.18 (1.10, 1.27)	**<0.001**	1.25 (1.16, 1.35)	**<0.001**	0.271
Jalisco	1.12 (1.07, 1.17)	**<0.001**	1.16 (1.10, 1.21)	**<0.001**	0.297
Mexico	1.11 (1.08, 1.15)	**<0.001**	1.17 (1.32, 1.21)	**<0.001**	**0.032**
Michoacán	1.11 (1.07, 1.17)	**<0.001**	1.16 (1.10, 1.22)	**<0.001**	0.254
Morelos	1.09 (1.00, 1.19)	0.063	1.25 (1.13, 1.37)	**<0.001**	**0.034**
Nayarit	1.13 (1.00, 1.28)	0.051	1.21 (1.06, 1.39)	**0.004**	0.423
Nuevo Leon	1.19 (1.13, 1.27)	**<0.001**	1.33 (1.25, 1.42)	**<0.001**	**0.015**
Oaxaca	1.15 (1.09, 1.22)	**<0.001**	1.21 (1.13, 1.29)	**<0.001**	0.346
Puebla	1.13 (1.09, 1.18)	**<0.001**	1.18 (1.13, 1.24)	**<0.001**	0.165
Querétaro De Arteaga	1.12 (1.04, 1.22)	**0.005**	1.07 (0.98, 1.16)	0.134	0.400
Quintana Roo	1.16 (1.01, 1.34)	**0.038**	1.27 (1.10, 1.49)	**0.003**	0.438
San Luis Potosi	1.18 (1.11, 1.26)	**<0.001**	1.16 (1.08, 1.24)	**<0.001**	0.653
Sinaloa	1.26 (1.17, 1.36)	**<0.001**	1.29 (1.19, 1.39)	**<0.001**	0.678
Sonora	1.28 (1.18, 1.39)	**<0.001**	1.27 (1.16, 1.39)	**<0.001**	0.920
Tabasco	1.14 (1.06, 1.22)	**<0.001**	1.20 (1.11, 1.30)	**<0.001**	0.294
Tamaulipas	1.18 (1.09, 1.27)	**<0.001**	1.16 (1.07, 1.25)	**<0.001**	0.763
Tlaxcala	1.22 (1.10, 1.35)	**<0.001**	1.23 (1.11, 1.36)	**<0.001**	0.914
Veracruz de Ignacio de La Llave	1.16 (1.11, 1.21)	**<0.001**	1.25 (1.19, 1.31)	**<0.001**	**0.028**
Yucatan	1.18 (1.10, 1.27)	**<0.001**	1.20 (1.11, 1.30)	**<0.001**	0.782
Zacatecas	1.12 (1.04, 1.21)	**0.002**	1.37 (1.26, 1.49)	**<0.001**	**0.001**

*Significant p value <0.05.

**Interaction sex(male and female)*year (2008 and 2017); significant p value<0.10.

LBW incidence at the municipal level reflects important disparities (S2a–S2d Fig). In the study period, the number of municipalities with LBW ≥8% increased from 460 to 700 in males and from 349 to 588 in females. In 2008, there were 11 municipalities with an incidence of LBW ≥50% in males (9 in Oaxaca and 2 in Veracruz) and 12 municipalities in females (11 in Oaxaca and 1 in Chihuahua). The number of recorded births in those municipalities was very low (1 to 6 births). In contrast, in 2017, there were 7 municipalities with an incidence of LBW ≥ 50% in males (6 in Oaxaca and 1 in Chiapas) and 9 in females (8 in Oaxaca and 1 in Nuevo León). These municipalities had a range of 1 to 71 recorded births. Mexico City, the state zoomed in on (S2a–S2d Fig) reported an important increase in LBW with a higher prevalence of female newborns.

### LBW incidence according to maternal characteristics

LBW incidence in both females and males increased from 2008 to 2017 regardless of maternal characteristics (Table 2), except in newborn males whose mothers were divorced, widows, or separated in which LBW incidence remained the same. The highest incidence of LBW in both years occurred in newborns from women over 50 years old and for those whose mothers did not receive prenatal care.

**Table 2:** Incidence of LBW according to maternal characteristics, 2008 and 2017.

	2008						2017					
	Male		Female		Both		Male		Female		Both	
	N	%	N	%	N	%	N	%	N	%	N	%
**Age**												
<18	134,921	7.1	124,212	6.3	259,133	6.7	138,112	7.9	127,318	7.2	265,430	7.6
19 to 34	726,483	6.0	685,376	5.7	1,411,859	5.9	769,006	6.4	723,001	6.6	1,492,007	6.7
35 to 49	84,394	8.0	81,336	7.3	165,730	7.6	97,816	9.6	93,494	9.2	191,310	9.4
≥50	394	7.6	328	9.1	722	8.3	78	38.5	61	29.5	139	34.5
**Education**												
Less than elementary	107,205	6.2	100,447	5.5	207,652	5.8	53,202	7.5	49,147	6.9	102,349	7.2
Elementary	226,421	6.2	212,846	5.6	439,267	5.9	137,997	7.2	128,269	6.7	266,266	7.0
Secondary	346,612	6.3	326,540	5.8	673,152	6.1	397,593	7.1	373,719	6.8	771,312	6.9
Complete high school	149,566	6.5	141,245	6.2	290,811	6.3	255,699	6.9	240,282	6.7	495,981	7.0
Bachelor degree	109,224	7.1	103,237	7.1	212,461	7.1	147,019	7.9	139,750	8.1	286,769	8.0
**Health care provider**												
IMSS	261,804	6.5	248,602	6.3	510,406	6.4	264,931	7.5	250,633	7.5	515,564	7.5
ISSSTE	22,786	7.4	21,997	7.0	44,783	7.2	28,085	8.3	26,952	8.5	55,037	8.4
PEMEX	2,850	6.3	2,882	7.0	5,732	6.7	2,108	8.8	2,075	7.8	4,183	8.3
SEDENA	6,074	8.1	5,599	6.4	11,673	7.3	5,131	7.2	4,801	7.5	9,932	7.3
SEMAR	1,825	5.9	1,739	4.2	3,564	5.1	1,090	7.2	1,028	5.5	2,118	6.4
Seguro Popular	219,061	5.7	206,089	5.1	425,150	5.4	515,100	7.0	481,319	6.6	996,419	6.8
IMSS Oportunidades	Not reported*						40,674	5.3	38,274	5.0	78,948	5.2
Other	30,129	6.4	28,606	5.9	58,735	6.2	40,361	8.7	38,247	8.2	78,608	8.2
**Marital status**												
Married	483,527	6.2	458,626	5.9	942,153	6.1	364,862	7.2	345,735	7.1	710,597	7.1
Singled	93,306	7.2	87,244	6.6	180,550	6.9	96,111	7.9	88,711	7.8	184,822	7.9
Divorced/widow/separated	3,296	7.4	3,173	6.0	6,469	6.7	5,292	7.4	4,942	8.0	10,234	7.7
Consensual union	357,257	6.3	333,874	5.9	691,131	6.1	515621	7.07	482,653	6.7	998274	6.9
**Area of residency**												
Suburban and urban	587,984	6.3	554,567	5.9	1,142,551	6.1	726,264	7.3	682,725	7.1	1,408,989	7.2
Rural	144,654	5.3	135,691	4.8	280,345	5.1	151,779	6.3	141,586	5.9	293,365	6.1
**Marginalization index**												
Very low	311,578	6.5	295,071	6.2	606,649	6.4	399,621	7.5	377,453	7.4	777,074	7.5
Low	161,222	6.2	151,589	5.7	312,811	6.0	185,358	7.2	174,170	6.9	359,528	7.1
Medium	95,082	5.7	89,530	5.3	184,612	5.5	101,496	6.8	94,968	6.4	196,464	6.6
High	154,322	5.5	144,477	4.9	298,799	5.2	175,196	6.5	162,880	6.0	338,076	6.2
Very high	10,434	5.4	9,591	4.1	20,025	4.8	16,372	6.1	14,840	5.8	31,212	6.0
**Parity**												
1	342,646	6.9	316,261	6.25	658,907	6.6	366,465	7.7	338,343	7.2	704,808	7.4
2	272,375	5.9	257,518	5.7	529,893	5.8	310,426	6.6	293,225	6.6	603,651	6.6
≥3	337,322	6.2	323,067	5.8	660,389	6.0	328,366	7.2	312,523	7.1	640,889	7.2
**Prenatal care**												
Yes	914,262	6.3	861,600	5.9	177,5862	6.1	981,821	7.1	922,651	6.9	1,904,472	7.0
No	32,248	9.0	29,638	8.2	61,886	8.6	20,709	11.4	18,845	10.3	39,554	10.7
**First prenatal visit**												
None												
First trimester	662,648	6.4	628,174	6.0	1,290,822	6.2	768,444	7.1	724,870	6.9	1,493,314	7.0
Second trimester	194,750	6.2	181,060	5.7	375,810	5.9	174,559	7.1	161,465	6.6	336,024	6.9
Third trimester	45,001	5.8	41,705	5.3	86,706	5.5	30,590	6.7	28,635	6.6	59,225	6.7
Term	885,268	2.7	838,889	2.4	1,724,157	2.6	933,411	2.9	881,835	2.8	1,815,246	2.9

Abbreviations: IMSS, Instituto Mexicano del Seguro Social; ISSSTE, Instituto de Seguridad y Servicios Sociales de los Trabajadores del Estado; PEMEX, Petroleos mexicanos; SEDENA, Secretaría de la Defensa Nacional; SEMAR, Secretaría de Marina.

* Not reported because in 2008 this program did not exist.

** In some cases the birth records had incomplete maternal information. Therefore, the sample size according to maternal characteristics is different.

## Discussion

To our knowledge, this is the first study that reports the incidence and trends of LBW in Mexico, at the national, state and municipality levels. The results from this study are relevant for tracking the progress towards reducing this public health concern in the country as well as identifying high-risk groups to design and target interventions. The incidence of LBW in Mexico, at the national level, progressively increased from 6.2% in 2008 to 7.1% in 2017. At the state level, Mexico City reported the highest incidence followed by Southeastern and Central states (Mexico State, Aguascalientes and Yucatan); however, the states with the highest increase are located in the North of the country (Baja California Sur, Nuevo León, and Zacatecas). At the municipal level, there is no clear geographic pattern observed in the incidence of LBW. Municipalities with incidences ≥8% were distributed throughout the country. We observed that the incidence of LBW was higher in urban areas, in places with low indices of marginalization, and among mothers ≥35 years old and without prenatal care. Similarly, other studies have identified that high maternal age and inadequate prenatal care are associated with LBW [8, 27, 28]. Despite Mexican legislation mandates that pregnant women with low risk for complications during gestation should have at least five prenatal visits starting as early as between the 6^th^ and 8^th^ week of pregnancy [15], by 2012, 15.7% of pregnant women did not receive prenatal attention during the first trimester of pregnancy [29].

Seguro Popular is a public health insurance that covers a wide range of services without co-pays for its affiliates [30]. It was established by the Mexican government in an effort to expand health care to those without insurance (mainly poor families) and reduce health inequities. Although Seguro Popular coverage increased 28.2 pp in the study period, prenatal care only increased 1.4 pp. It is crucial to explore further the reasons for this lack of prenatal care to develop public health programs to prevent LBW. For instance, a communication intervention that included a poster, a calendar, a brochure, and two radio songs was developed for low-income pregnant women in the northeast of Mexico to encourage the use of prenatal care, provide information about gain weight and good nutrition and improve women’s skills to prevent complications [31]. This type of knowledge translation intervention for prenatal care need to be developed and tested to increase education among pregnant women and subsequently, reduce the incidence of LBW.

The Mexican incidence of LBW was similar to the incidence reported for Latin America (8.7%) by WHO [1]. The increase of 0.9 pp in our studied period of follow-up represents an increase of 14.6% in the LBW incidence and implies an increase of 900 cases per 100,000 births per year, increasing the number of cases expected for the following years by 7,100 cases per 100,000 births. This increase is relevant due to the short and long-term consequences that LBW has on individual health, as well as the associated economic costs for families and the health system [32].

According to UNICEF estimations [33], a reduction in the prevalence of LBW was observed, both worldwide and in the Latin America region, during the period from 2000 to 2015. Similarly, Mexican prevalences reduced, they were 8.2, 8.0, and 7.9 in the years 2000, 2012, and 2015, respectively. Nevertheless, our study’s results contrast with these results since we found the incidence of LBW in Mexico increased from 2008 to 2017. Another study conducted in Mexico by Buekens et al. [19] using SINAIS and CONAPO databases also estimated an increase in the prevalence of LBW from 2008 to 2009 (8.2% and 8.5%, respectively). Despite our results coincide with the increase in LBW with this study [19], we observed a lower incidence in 2008 (6.2%) than the one reported in the aforementioned study. In other Latin American middle-income countries such as Colombia [34] and Brazil [35] an increase in the incidence of LBW has also been observed.

Mexican states with the highest incidences of LBW are located in Southeastern and Central Mexico. These regions are characterized by higher rates of poverty [36] and high and medium marginalization indexes [22] which indicated the interconnection between LBW, poverty and socioeconomic factors. The Northern states (Baja California Norte, Sonora) had lower incidences of LBW and this geographic area had few poverty levels. Another explanation could be the maternal height, this variable has been identified as a birth weight predictor [37] and people who lived in the Northern are taller than people in the Southern [38].

Mexico City and localities with very low marginalized index and higher rates of LBW incidence deserve special attention, it seems contradictory that places with better living conditions have higher incidences of LBW. In the case of Mexico City, although it has a lower marginalization index [22] compared with the rest of the country, it presented the highest incidence throughout the studied period in both newborn females and males. A possible explanation is that in Mexico City most births occur in hospitals, therefore, few births are non-reported. It is likely that other states where more births occur outside the hospitals, which are usually non-reported, are underestimating their LBW incidence. This situation is common in LMICs where data from vulnerable populations, is usually limited and unreliable which may underestimate LBW incidence [8]. Another plausible explanation is the association between preterm birth and LBW [39]. Women in urban areas are at higher risk of preterm delivery which is the most common cause of LBW [40]. Moreover, studies have found that in populations with a higher quality of life, the incidence of LBW is higher. For example, a study conducted in Brazil [35] observed that the LBW rate was higher in more developed regions than in less developed regions and a similar trend was observed in Pennsylvania in the United States [40].

It is important to notice that in some municipalities the incidence of LBW was higher than 50%. In most of these, the number of recorded births ranged from 1 to 9 births. This situation caused the estimated incidences of LBW to be very high but reflects the reality of these municipalities [41]. It is important to identify the municipalities most affected by LBW even if the number of births is low, so that prevention programs can be tailored to those areas.

Important limitations need to be considered when analyzing the results from this study. The databases we use may not cover all the births that occurred in the country. In Mexico, it is estimated that 89.3% of births occur in hospitals, 3.8% in homes, and approximately 6% of births occur in unspecified places. It is not possible to know if all births occurring outside hospitals are registered in SINAC. In addition, errors in measurement and recording of birth weight, as well as, the misclassification between live births and stillbirths, are also inherent limitations to the data we used. The localities with the highest vulnerability are those that could have a higher under-registration of births and therefore the incidences of LBW that we reported could be underestimating this public health problem.

Another important limitation is the lack of maternal anthropometric data (e.g. height and Body Mass Index) and other variables such as the altitude, complications during pregnancy and type of delivery that can be related to LBW.

Despite these limitations, the results of this study represent the first effort to systematize the LBW statistics at different levels of geographic disaggregation. It is critical to invest in a report system that keeps track of this important health indicator, this implies investing both in the infrastructure and in the training of the personnel who participate in the measurements, recording and systematization of this indicator. The results from this study may assist government entities, researchers and stakeholders in the design and implementation of appropriate interventions, community-based programs, and public policies that aim to improving maternal and child health indicators. The regionalization at state and municipal levels of LBW incidence allowed us to identify the most vulnerable areas in the country and analyze the maternal and sociodemographic characteristics that may be related to LBW incidence. The spatial data analysis is an important tool that facilitates the visualization of the states and municipalities more affected.

## Conclusion

Mexico faces a complex situation to meet the goals proposed by the WHO for 2025. Future studies should aim to identify the determinants of health associated with LBW, to design and implement community-based programs and public policies targeted to reverse the trend and achieve the goals proposed by the WHO in the World Nutrition Goals 2025.

## Supporting information

S1 Table Maternal and newborn characteristics in 2008 and 2017. (PDF)

S1 Fig Mean birth weight distribution and change between 2008 and 2017 by sex. (TIF)

S2 Fig LBW incidence in live births in Mexico at the municipal level in males and females in 2008 and 2017. (TIF)

## References

[1] WHO. Global Nutrition Targets 2025: Low birth weight policy brief. WHO. 2018.

[2] Cutland CL, Lackritz EM, Mallett-Moore T, Bardají A, Chandrasekaran R, Lahariya C, et al. Low birth weight: Case definition & guidelines for data collection, analysis, and presentation of maternal immunization safety data. Vaccine. 2017 Dec 4;35(48Part A):6492–500. 29150054 10.1016/j.vaccine.2017.01.049 PMC5710991

[3] Kramer MS. Determinants of low birth weight: methodological assessment and meta-analysis. Bull World Health Organ. 1987;65(5):663–737. 3322602 PMC2491072

[4] Lira PI, Ashworth A, Morris SS. Low birth weight and morbidity from diarrhea and respiratory infection in northeast Brazil. J Pediatr. 1996 Apr;128(4):497–504. doi: 10.1016/s0022-3476(96)70360-8 8618183

[5] Hack M, Klein NK, Taylor HG. Long-term developmental outcomes of low birth weight infants. Future Child. 1995;5(1):176–96. 7543353

[6] Zerbeto AB, Cortelo FM, Filho ÉBC. Association between gestational age and birth weight on the language development of Brazilian children: a systematic review. J Pediatr (Rio J). 2015 Aug;91(4):326–32. doi: 10.1016/j.jped.2014.11.003 25913048

[7] UNICEF. WHO. Low birthweight: country, regional and global estimates. New York 2004.

[8] Mahumud RA, Sultana M, Sarker AR. Distribution and Determinants of Low Birth Weight in Developing Countries. J Prev Med Public Health. 2017 Jan;50(1):18–28. doi: 10.3961/jpmph.16.087 28173687 PMC5327679

[9] Blencowe H, Krasevec J, de Onis M, Black RE, An X, Stevens GA, et al. National, regional, and worldwide estimates of low birthweight in 2015, with trends from 2000: a systematic analysis. The Lancet Global Health. 2019 Jul 1;7(7):e849–60. doi: 10.1016/S2214-109X(18)30565-5 31103470 PMC6560046

[10] Nyirenda MJ, Byass P. Pregnancy, programming, and predisposition. The Lancet Global Health. 2019 Apr 1;7(4):e404–5. doi: 10.1016/S2214-109X(19)30051-8 30799145

[11] Martin JA, Hamilton BE, Osterman MJK, Driscoll AK. National Vital Statistics Reports Volume 70, Number 2, March 23 Births: Final Data for 2019. 2019;51. 33814033

[12] WHO. Global nutrition targets 2025: policy brief series (WHO/NMH/NHD/14.2). Geneva: World Health Organization; 2014.

[13] INEGI. División territorial. Cuéntame de México [Internet]. Mexico: INEGI [cited 2021 Apr 5]. http://cuentame.inegi.org.mx/territorio/division/default.aspx?tema=T

[14] Subsistema de Información sobre Nacimientos (SINAC) [Internet]. Mexico: INEGI [cited 2021 Apr 5]. http://www.dgis.salud.gob.mx/contenidos/sinais/s_sinac.html (accessed on 25 June 2020).

[15] Secretaría de Salud. Norma Oficial NOM-007-SSA2-2016, para la atención de la mujer durante el embarazo, parto y puerperio, y de la personal recién nacida. México, 2016.

[16] Rodríguez Guzmán LM, Romero Tinoco P, Andrade García M, Velázquez Luna M, Rodríguez García R. Prevalence of low weight at birth and related factors. Ginecol Obstet Mex. 2005 Mar;73(3):132–6. 21961351

[17] Lezama Hernández MP, Díaz Gómez JM, Rodríguez Zetina R. Prevalencia de bajo peso al nacimiento en un Hospital General de segundo nivel. Salud en Tabasco. 2001;7(2):401–3.

[18] Frank R, Pelcastre B, Salgado de Snyder VN, Frisbie WP, Potter JE, Bronfman-Pertzovsky MN. Low birth weight in Mexico: new evidence from a multi-site postpartum hospital survey. Salud Publica Mex. 2004 Feb;46(1):23–31. doi: 10.1590/s0036-36342004000100004 15053393

[19] Buekens P, Canfield C, Padilla N, Lara Lona E, Lozano R. Low birthweight in Mexico: a systematic review. Matern Child Health J. 2013 Jan;17(1):129–35. doi: 10.1007/s10995-012-0956-4 22322429

[20] Nacimientos datos abiertos [Internet]. Dirección General de Información en Salud, Secretaría de Salud. [cited 2019 Aug 26]. http://www.dgis.salud.gob.mx/contenidos/basesdedatos/da_nacimientos_gobmx.html

[21] Dirección General de Información en Salud. Secretaría de Saluds. Manual de Llenado del Certificado de Nacimientos [Internet]. Mexico; 2015. http://www.dgis.salud.gob.mx/descargas/pdf/CN_ManualLlenado.pdf

[22] Datos abiertos del índice de marginación [Internet]. Consejo Nacional de Población (CONAPO). [cited 2019 Aug 26]. http://www.conapo.gob.mx/es/CONAPO/Datos_Abiertos_del_Indice_de_Marginacion.

[23] Población. Rural y urbana [Internet]. Mexico: INEGI [cited 2020 Oct 3]. http://cuentame.inegi.org.mx/poblacion/rur_urb.aspx?tema=P

[24] CONAPO. Índice absoluto de marginación 2000–2010. Mexico: CONAPO [cited 2020 Oct 3]. http://www.conapo.gob.mx/es/CONAPO/Indice_Absoluto_de_Marginacion_2000_2010

[25] INEGI. Marco Geoestadístico Nacional. Mexico: INEGI [cited 2020 Oct 3]. https://www.inegi.org.mx/temas/mg/.

[26] QGIS Development Team, 2019. QGIS Geographic Information System. Open Source Geospatial Foundation Project. https://www.qgis.org/en/site/.

[27] Pinzón-Rondón ÁM, Gutiérrez-Pinzon V, Madriñan-Navia H, Amin J, Aguilera-Otalvaro P, Hoyos-Martínez A. Low birth weight and prenatal care in Colombia: a cross-sectional study. BMC Pregnancy Childbirth. 2015 May 20;15:118. doi: 10.1186/s12884-015-0541-0 25989797 PMC4491421

[28] Goisis A, Remes H, Barclay K, Martikainen P, Myrskylä M. Advanced Maternal Age and the Risk of Low Birth Weight and Preterm Delivery: a Within-Family Analysis Using Finnish Population Registers. Am J Epidemiol. 2017 Dec 1;186(11):1219–26. doi: 10.1093/aje/kwx177 29206985 PMC5860004

[29] Instituto Nacional de Salud Pública, 2012. Encuesta Nacional de Salud y Nutrición 2012. Resultados Nacionales. Síntesis Ejecutiva. Cuernavaca, Morelos, México.

[30] Seguro Popular: Health Coverage For All in Mexico [Internet]. World Bank. [cited 2021 Jul 15]. https://www.worldbank.org/en/results/2015/02/26/health-coverage-for-all-in-mexico

[31] Alcalay R, Ghee A, Scrimshaw S. Designing prenatal care messages for low-income Mexican women. Public Health Rep. 1993;108(3):354–62. 8497574 PMC1403387

[32] Thanh NX, Toye J, Savu A, Kumar M, Kaul P. Health Service Use and Costs Associated with Low Birth Weight—A Population Level Analysis. J Pediatr. 2015 Sep;167(3):551–556.e1-3. doi: 10.1016/j.jpeds.2015.06.007 26148659

[33] United Nations Children’s Fund (UNICEF), World Health Organization (WHO). UNICEF-WHO Low birthweight estimates: Levels and trends 2000–2015. Geneva: World Health Organization; 2019 Licence: CC BY-NC-SA 3.0 IGO.

[34] Márquez-Beltrán Marlon F. R., Vargas-Hernández Jhonny E., Quiroga-Villalobos Edwin F., et al. (2013). Análisis del bajo peso al nacer en Colombia 2005–2009. Revista de Salud Pública, 15(4), 626–637. 25124129

[35] da Silva AAM, da Silva LM, Barbieri MA, Bettiol H, de Carvalho LM, Ribeiro VS, et al. The epidemiologic paradox of low birth weight in Brazil. Revista de Saúde Pública. 2010 Oct;44(5):767–75. doi: 10.1590/s0034-89102010005000033 20835496

[36] Consejo Nacional de Evaluación de la Política de Desarrollo Social (CONEVAL). “Medición de la pobreza, Estados Unidos Mexicanos, serie 2008–2018”. [Internet]. https://www.coneval.org.mx/Medicion/Paginas/PobrezaInicio.aspx.

[37] Inoue S, Naruse H, Yorifuji T, Kato T, Murakoshi T, Doi H, et al. Association between Short Maternal Height and Low Birth Weight: a Hospital-based Study in Japan. J Korean Med Sci. 2016 Mar;31(3):353–9. doi: 10.3346/jkms.2016.31.3.353 26955234 PMC4779858

[38] Castro-Porras LV, Rojas-Russell ME, Aedo-Santos Á, Wynne-Bannister EG, López-Cervantes M. Stature in adults as an indicator of socioeconomic inequalities in Mexico. Rev Panam Salud Publica. 2018;42:e29. doi: 10.26633/RPSP.2018.29 31093058 PMC6386041

[39] World Health Organization. Guidelines on optimal feeding of low birth-weight infants in low- and middle-income countries. 2011. 26042325

[40] Hillemeier MM, Weisman CS, Chase GA, Dyer A-M. Individual and community predictors of preterm birth and low birthweight along the rural-urban continuum in central Pennsylvania. J Rural Health. 2007;23(1):42–8. doi: 10.1111/j.1748-0361.2006.00066.x 17300477

[41] National Council for the Evaluation of Social Development Policy. Report of Poverty in Mexico 2010: The Country, Its States and Its Municipalities. Mexico, Federal District, CONEVAL, 2012.

