# Supplementary material for: Incidence of low birth weight in Mexico: A descriptive retrospective study from 2008–2017
Source: PLoS One. 2021 Sep 10;16(9):e0256518. doi: 10.1371/journal.pone.0256518 (PMC8432805; doi:10.1371/journal.pone.0256518)
Supplement: S1 Table — (PDF) [file pone.0256518.s001.pdf]

PLOS ONE

Incidence of low birth weight in Mexico: a descriptive retrospective study from 2008 - 2017

Mónica Ancira-Moreno, Eric Monterubio-Flores, Sonia Hernández-Cordero, Isabel Omaña-Guzmán, Isidro Soloaga, Fabián Torres, Moisés Reyes, Yohali Burrola-Mendez, and Arianna Morales-López.

Supporting Table 1. Maternal and newborn characteristics in 2008 and 2017

	2008		2017	
**Maternal information**				
	**N**	**%**	**N**	**%**
**Age**				
<18	259,132	14.1	265,430	13.6
19 to 34	1,411,859	76.8	1,492,007	76.6
35 to 49	165,730	9.0	191,310	9.8
≥50	722	0.04	139	0.01
**Education**				
Less than elementary	207,652	11.4	102,349	5.3
Elementary	439,267	24.1	266,266	13.9
Secondary	673,152	36.9	771,312	40.1
High school	290,811	16.0	495,981	25.8
Bachelor degree or more	212,461	11.7	286,769	14.9
**Health care provider**[1]				
IMSS	510,406	27.5	515,564	26.4
ISSSTE	44,783	2.4	55,037	2.8
PEMEX	5,732	0.3	4,183	0.2
SEDENA	11,673	0.6	9,932	0.5
SEMAR	3,564	0.2	2,118	0.1
Seguro Popular	425,150	22.9	996,419	51.1
IMSS Oportunidades	Not reported*	Not reported*	78,948	4.1
Other	58,735	3.2	78,608	4.0
**Marital status**				
Married	942,153	51.8	710,597	37.3

	2008		2017	
**Maternal information**				
Singled	180,550	9.9	184,822	9.7
Divorcee/widow/separated	6,469	0.4	10,234	0.5
Consensual union	691,131	38.0	998,274	52.4
**Area of residency**				
Urban	1,304,098	80.7	1,542,171	82.5
Rural	311,656	19.3	326,506	17.5
**Marginalization index[2]**				
Very low	716,196	44.3	827,539	44.3
Low	374,485	23.2	440,145	23.6
Medium	197,256	12.2	212,734	11.4
High	308,817	19.1	358,348	19.2
Very high	19,000	1.2	29,911	1.6
**Prenatal care**				
Yes	1,775,862	96.6	1,904,472	98.0
No **	61,886	3.4	39,554	2.0
**First prenatal visit**				
None **	61,892	3.4	39,087	2.0
First trimester	1,290,822	71.1	1,493,314	77.5
Second trimester	376,810	20.7	336,024	17.4
Third trimester	86,706	4.8	59,225	3.1
Single pregnancy	1,816,589	98.7	1,916,488	98.5
Twin pregnancy	24,204	1.3	28,374	1.5
Multiple pregnancies (≥3)	818	0.04***	774	0.04***
**Newborn information**				
Male	954,570	51.5	1,005,460	51.6
Female	898,999	48.5	944,280	48.4
**Gestational age at birth[3]**				
Term	1,724,157	93.9	1,815,246	03.2
Preterm birth	111,076	6.1	132,681	6.8
Post-term birth	Not reported	Not reported	159	0.01***
	**N**	**Mean ± SD**	**N**	**Mean ± SD**
Birth weight	1,853,569	3179.3 ± 468.1	1,949,740	3135.8 ± 469.1
Length at birth	1,848,817	50.2 ± 3.0	1,910,605	49.9 ± 2.5

Abbreviations: SD, standard deviation; IMSS, Instituto Mexicano del Seguro Social; ISSSTE, Instituto de Seguridad y Servicios Sociales de los Trabajadores del Estado; PEMEX, Petroleos mexicanos; SEDENA, Secretaría de la Defensa Nacional; SEMAR, Secretaría de Marina.

Preterm birth (<37 weeks), term birth (37-41 weeks) and post term birth (>41 weeks).
*Not reported because in 2008 this program did not exist.
** There are missings data points on the prenatal care and first prenatal visit variables.
*** On these cases, two decimals were used.
