# Supplementary figures and images for: Incidence of low birth weight in Mexico: A descriptive retrospective study from 2008–2017
Source: PLoS One. 2021 Sep 10;16(9):e0256518. doi: 10.1371/journal.pone.0256518 (PMC8432805; doi:10.1371/journal.pone.0256518)

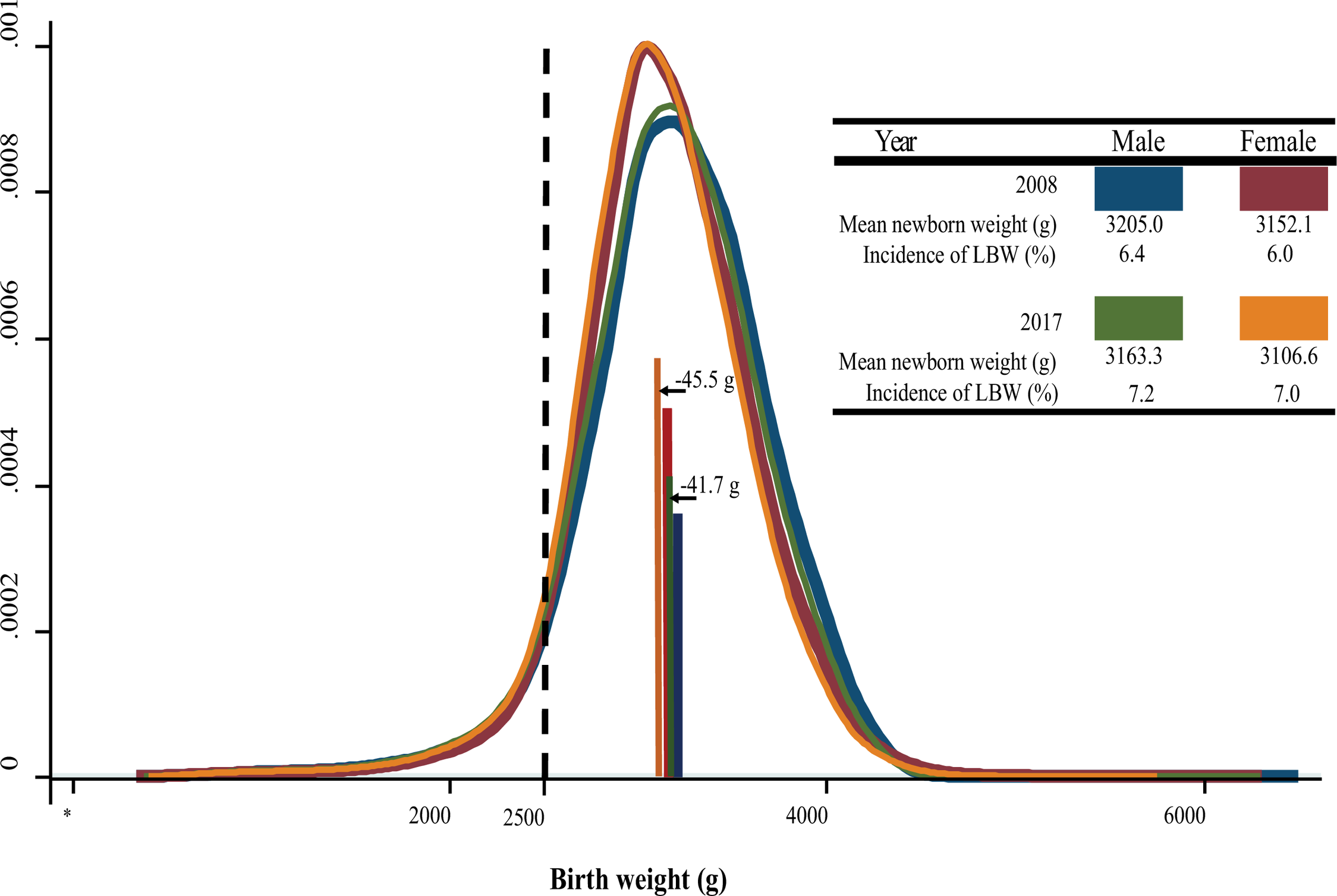

Supplement: S1 Fig — (TIF) [file pone.0256518.s002.tif]

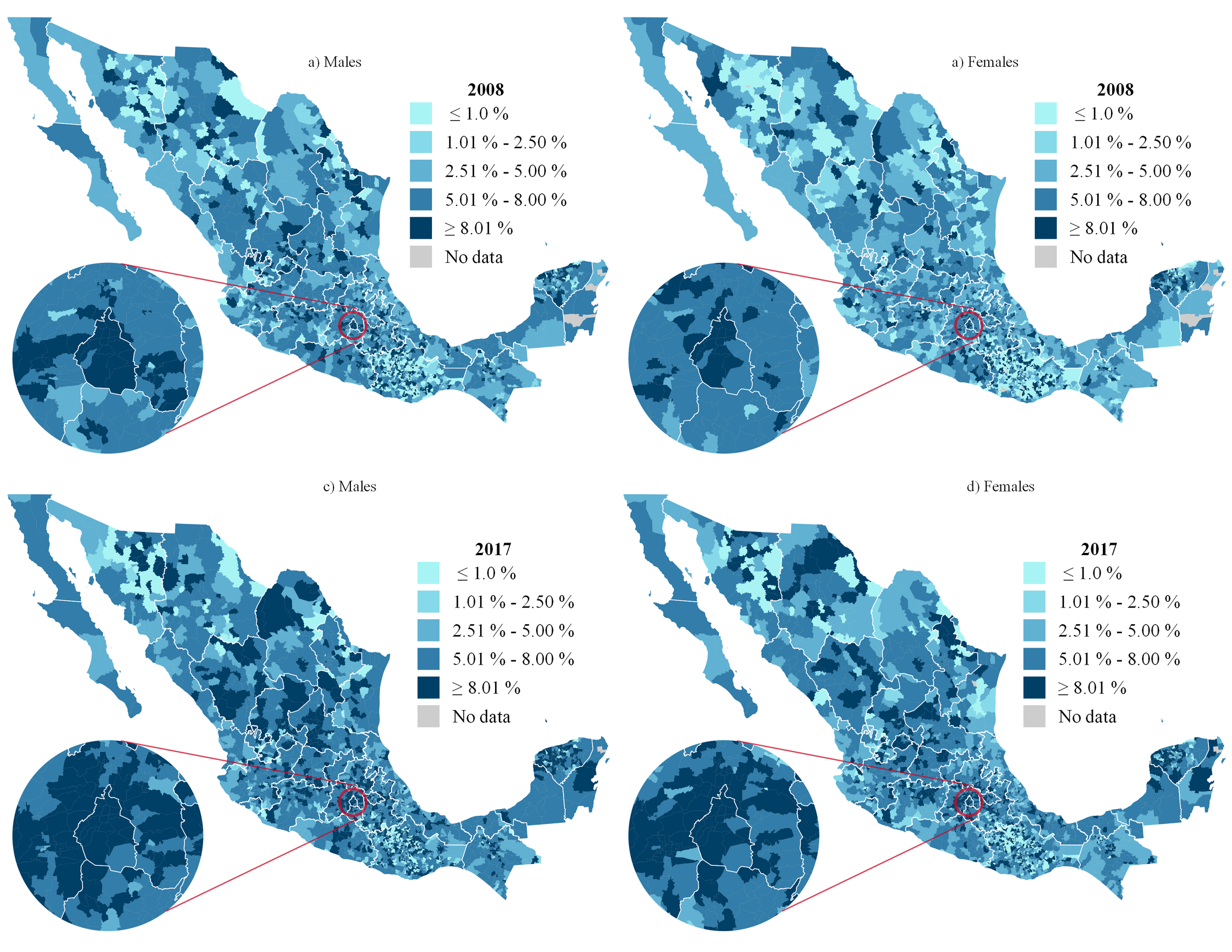

Supplement: S2 Fig — (TIF) [file pone.0256518.s003.tif]
